# Supplementary figures and images for: Cell type specific IL-27p28 (IL-30) deletion in mice uncovers an unexpected regulatory function of IL-30 in autoimmune inflammation
Source: Sci Rep. 2023 Feb 1;13:1812. doi: 10.1038/s41598-023-27413-w (PMC9892501; doi:10.1038/s41598-023-27413-w)

Supp Figure 1


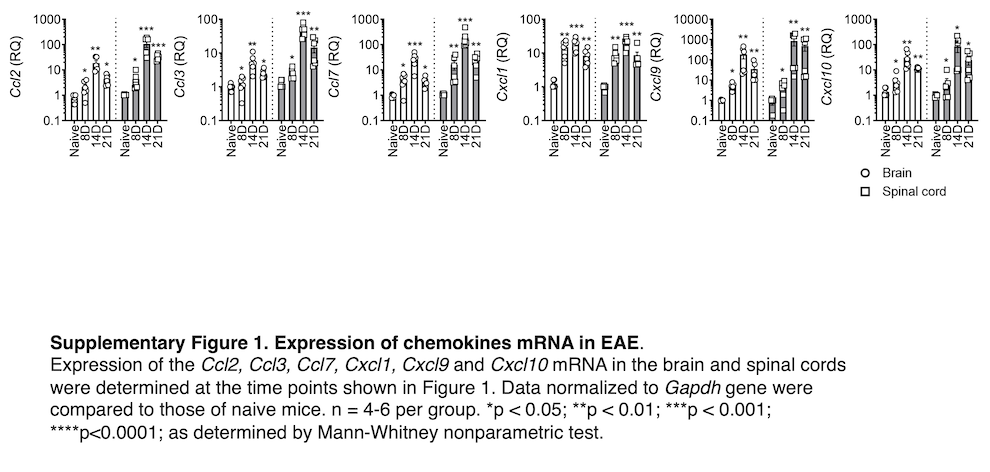


Supp Figure 2


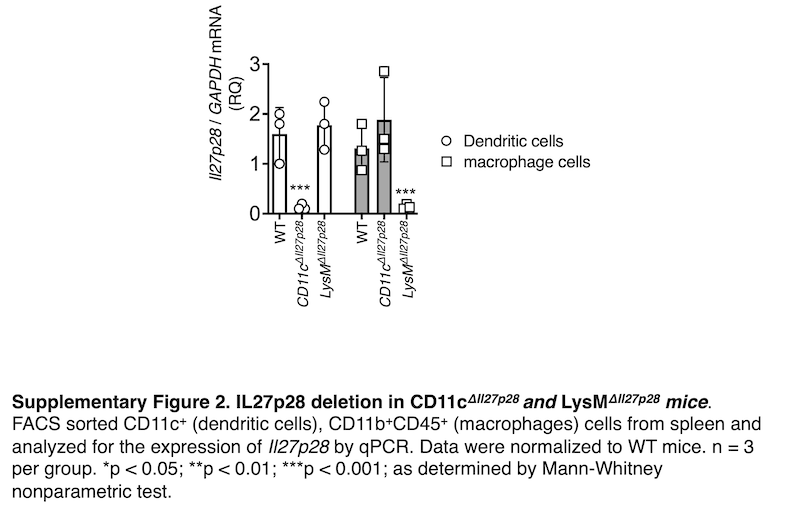


Supp Figure 3


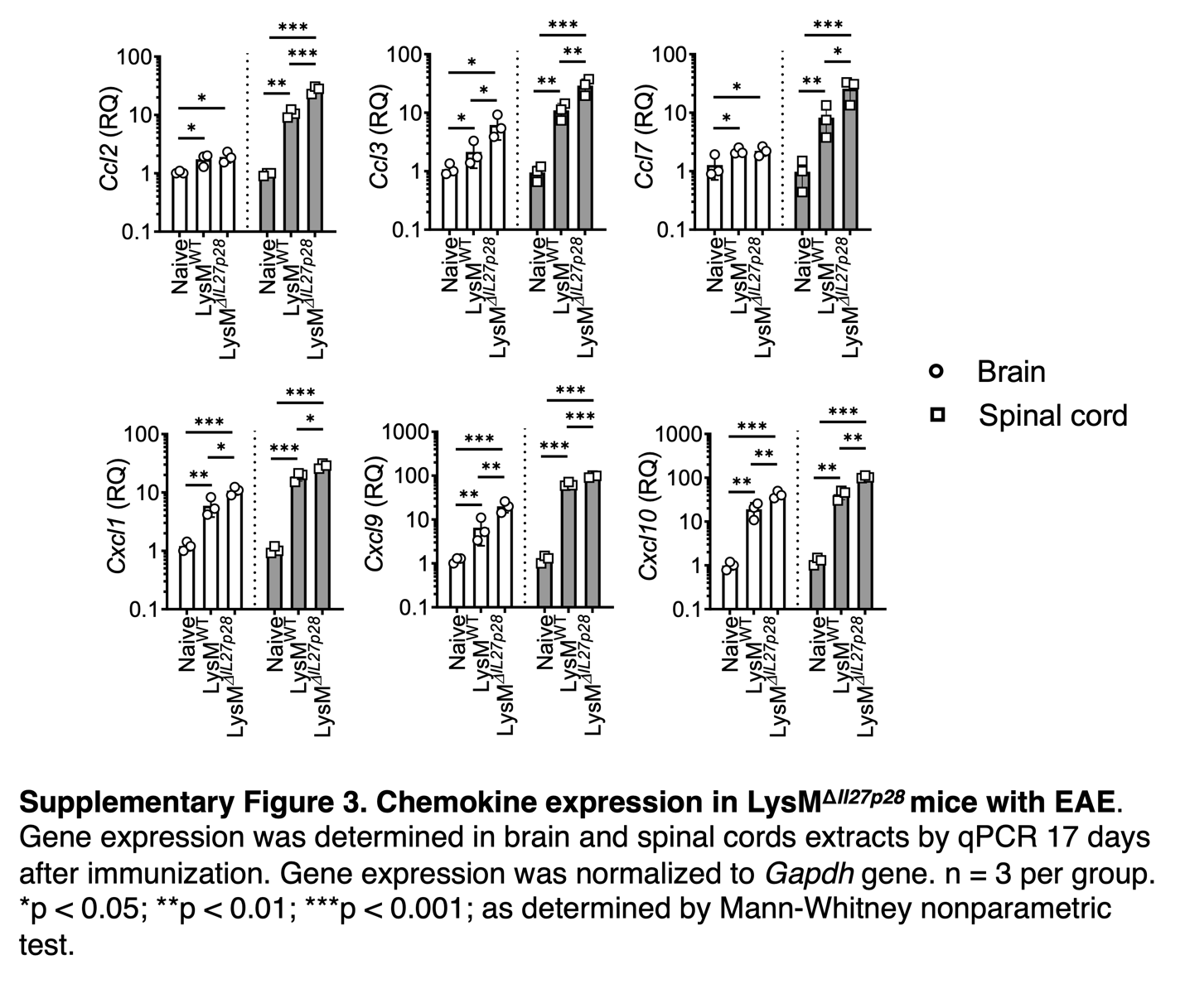


Supp Figure 4


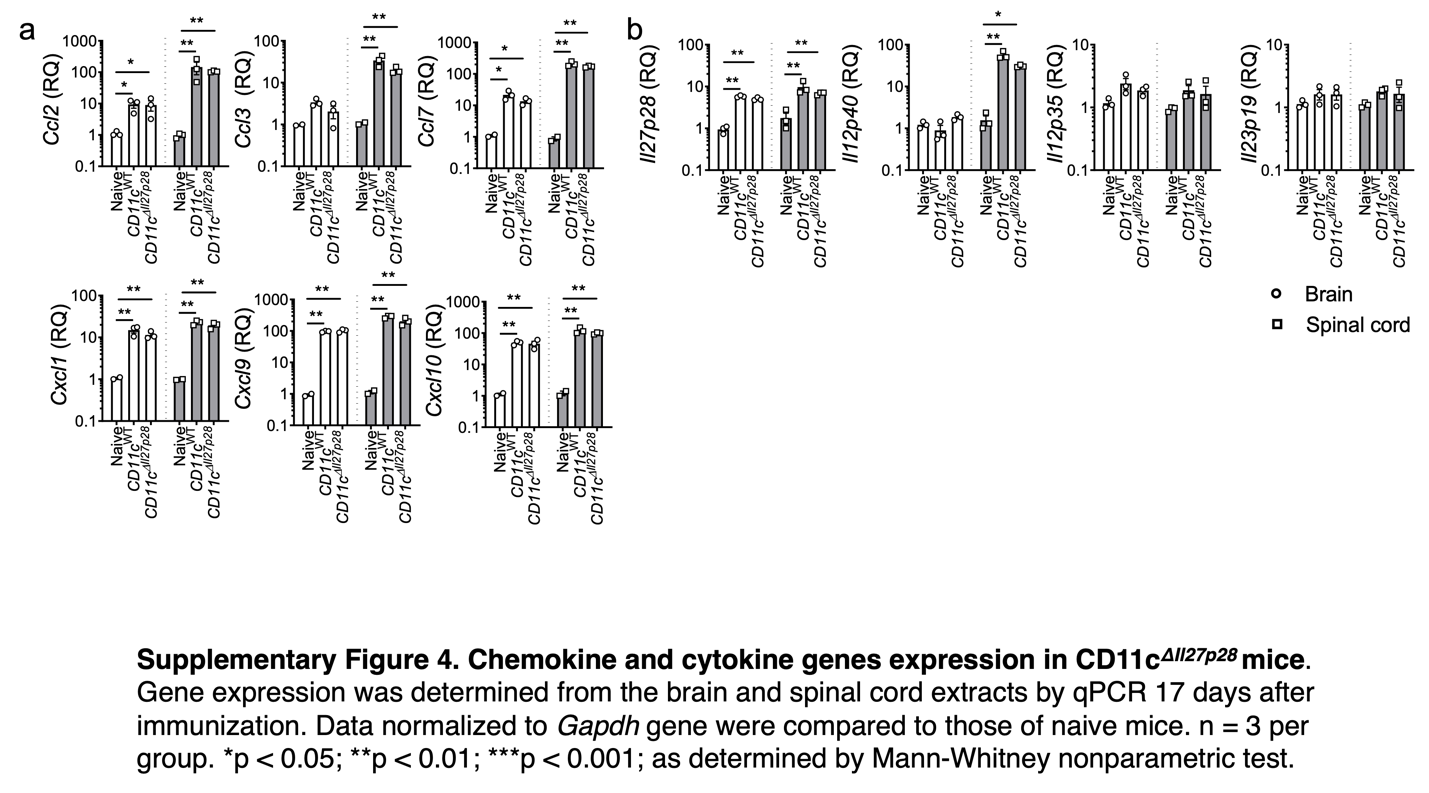


Supp Figure 5


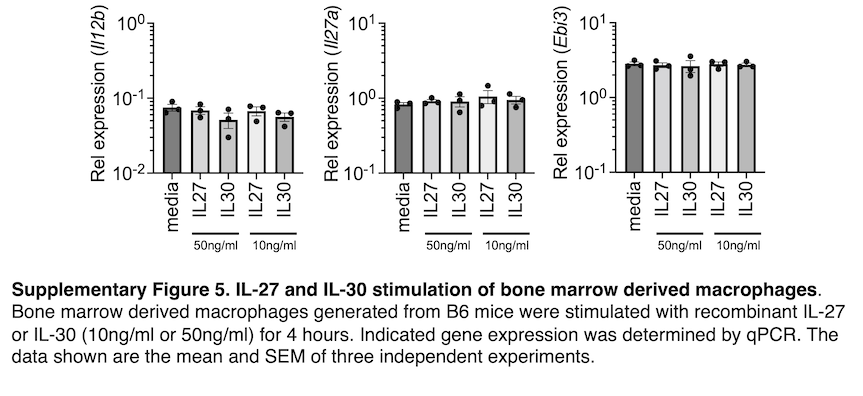


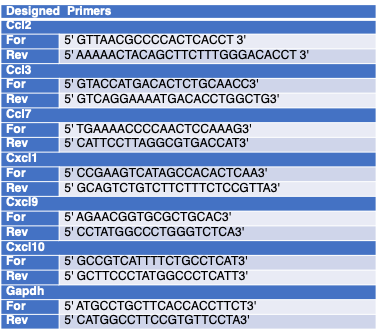
Supp Table 1

Supplement: Supplementary file 1 — Supplementary Information. [file 41598_2023_27413_MOESM1_ESM.docx]
